# *Acanthus montanus*: An experimental evaluation of the antimicrobial, anti-inflammatory and immunological properties of a traditional remedy for furuncles

**DOI:** 10.1186/1472-6882-8-27

**Published:** 2008-06-06

**Authors:** Charles O Okoli, Peter A Akah, Nkemjika J Onuoha, Theophine C Okoye, Anthonia C Nwoye, Chukwuemeka S Nworu

**Affiliations:** 1 Department of Pharmacology & Toxicology, Faculty of Pharmaceutical Sciences, University of Nigeria, Nsukka 410001, Enugu State, Nigeria

## Abstract

**Background:**

*Acanthus montanus *(Nees) T. Anderson (Acanthaceae) is a shrub widespread in Africa, the Balkans, Romania, Greece and Eastern Mediterranean. It is used in African traditional medicine for the treatment of urogenital infections, urethral pain, endometritis, urinary disease, cystitis, leucorrhoea, aches and pains. In southeastern Nigeria, the root is popular and acclaimed highly effective in the treatment of furuncles. This study was undertaken to experimentally evaluate the antimicrobial and anti-inflammatory properties of the root extract as well as its effect on phagocytosis and specific cell-mediated immune response which may underlie the usefulness of the roots in treatment of furuncles.

**Methods:**

The aqueous root extract (obtained by hot water maceration of the root powder) was studied for effects on the growth of clinically isolated strains of *Pseudomonas aeruginosa *and *Staphylococcus aureus*. The anti-inflammatory activity was investigated using acute topical edema of the mouse ear induced by xylene, acute paw edema induced by agar in rats, formaldehyde arthritis in rats, vascular permeability induced by acetic acid in mice and heat- and hypotonicity-induced haemolysis of ox red blood cells (RBCs). Also evaluated were the effects on *in vivo *leukocyte migration induced by agar, phagocytic activity of macrophages on *Candida albicans *and specific cell-mediated immune responses (delayed type hypersensitivity reaction (DTHR) induced by sheep red blood cell (SRBC)). The acute toxicity and lethality (LD_50_) in mice and phytochemical constituents of the extract were also determined.

**Results:**

The extract moderately inhibited the growth of the test organisms and significantly (*P *< 0.05) inhibited (57%) topical acute edema in the mouse ear. It significantly (*P *< 0.05) suppressed the development of acute edema of the rat paw in a non-dose-related manner and was not effective in inhibiting the global edematous response to formaldehyde arthritis. It also inhibited vascular permeability induced by acetic acid in mice and the haemolysis of ox RBCs induced by heat- and hypotonicity. The extract increased total leukocyte and neutrophil counts and caused a significant (*P *< 0.05) dose-related increase in the total number of macrophages at the 800 mg/kg dose. On phagocytic activity, the extract evoked a significant (*P *< 0.05) increase in the number of macrophages with ingested *C. albicans *at 800 mg/kg dose, and significantly (*P *< 0.05) inhibited DTHR in a dose-related manner. Phytochemical tests on the extract revealed an abundant presence of alkaloids and carbohydrates while saponins, glycosides, and terpenoids occurred in trace amounts. Acute toxicity test established an oral and intraperitoneal LD_50 _greater than 5,000 mg/kg.

**Conclusion:**

The effectiveness of the root of *A. montanus *in the treatment of furuncles may largely derive from mobilization of leukocytes to the site of the infection and activation of phagocytic activity as well as suppression of exacerbated immune responses by its constituents. Antimicrobial and anti-inflammatory activities are likely contributory mechanisms. Phytochemical constituents such as alkaloids and carbohydrates may be responsible for these pharmacological activities.

## Background

*Acanthus montanus *(Nees) T. Anderson (Acanthaceae), also known as "Bear's breeches", "Mountain thistle" or "Alligator plant", is a striking small shrub with sparse branches and soft stem. The morphology has been described [1]. It grows wild in grasslands, woods, scrub and rocky hills of the Balkans, Romania, Greece, Eastern Mediterranean and Africa [1].

*A. montanus *is popular in southern Nigeria where it is variously called "Elele-nyijuo", "Agamsoso" and "Agameru" and employed in traditional medicine [2]. In the Democratic Republic of Congo, the leaves are pounded in water with those of *Ananas comosus *and Costus spp and used to treat urogenital infections, urethral pain, endometritis, urinary disease, cystitis, leucorrhoea [3]. The roots are also used for bathing to relieve aches and pains [4]. Documented evidence of pharmacological activities shows that the leaves of the plant possess spasmolytic [5], analgesic [6], anti-inflammatory and antipyretic [7] properties. The isolation of constituents such as saponins [8] and the gammaceranes- acanthusol and its 3-O-β-D-glucopyranoside [9] from the plant has been documented.

In southeastern Nigeria, the root poultice is popularly used by the Igede people of Benue State [10] and Enugu-Ezike community of Enugu State to treat furuncles. Furuncle, also known as boil represents the commonest example of an abscess, and pyogenic organisms such as *Staphyloccocus aureus*, and *Pseudomonas aeruginosa *have been implicated as causal agents [11]. In local use, the root poultice is believed to cause "boil ripening", a lay expression for enhancement of pus accumulation in the boil thought to indicate that the infection has been surmounted. Pharmacologically however, pus accumulation is an evidence of infection by pyogenic micro organisms and activation of cellular immune responses. Infection by these organisms evokes the acute inflammatory response, causing tissue swelling and the associated pain [12] and leading to pyogenic or suppurative inflammation. In addition to pyogenic or suppurative inflammation, these bacteria produce potent exotoxins which affect all types of cells and promote considerable emigration of neutrophil polymorphs, resulting in formation of pus [11], a mixture of necrotic tissues, dead neutrophils and macrophages [13] the necrosis being a consequence of tissue digestion by neutrophils [11]. These cells contribute to non-specific defense of the body by phagocytosing and killing infecting organisms [14]. Discharge of pus from a ruptured boil restores the flow of exudates (earlier suppressed by pressure from accumulated pus) and hence the defense mechanisms leading to elimination of infection and commencement of healing [11]. From the pathophysiology, it is evident that the medicinal potency of an agent with such a popular use and acclaimed effectiveness in the treatment of furuncles may derive from direct inhibition of the growth of the causal organisms, suppression of acute inflammatory response and or enhancement of the host's ability to combat the infection through stimulation of immune responses. This study was undertaken to evaluate the effect of roots of this plant on the growth of causal organisms of furuncles and the inflammatory response as well as cell-mediated immune responses.

## Methods

### Plant material

Fresh roots of *A. montanus *were collected in February 2005 from Nsukka, Enugu State, Nigeria and authenticated by Mr. A. Ozioko of Bioresources Development and Conservation Program (BDCP) Centre, Nsukka where a voucher specimen (BDCP No. 308) is maintained. The plant material was cleaned, cut into smaller pieces, dried under the sun and reduced to a coarse powder using a Hammer Mill (Gallenkamp, USA). The powdered plant material (900 g) was extracted by macerating in hot water for 1 h. The extract was strained with a muslin cloth, allowed to cool and filtered. The filtrate was freeze dried to obtain 191.4 g (21.3% w/w) of aqueous extract (AE) which was subsequently subjected to phytochemical analysis using conventional methods [15].

### Pharmacological tests

#### Animals

Adult Swiss albino rats (150–250 g) and mice (15–30 g) of both sexes were obtained from the Laboratory Animal Facility of the Department of Pharmacology and Toxicology, Faculty of Pharmaceutical Sciences, University of Nigeria, Nsukka (UNN). Animals were housed in steel cages within the facility under standard conditions and allowed free access to standard pellets and water. Prior to their use, they were allowed two weeks for acclimatization within the work area environment. A healthy male sheep (for collection of sheep erythrocytes used for induction of DTHR) was taken from the herd at the Faculty of Veterinary Medicine, UNN. All animal experiments were in compliance with the National Institute of Health Guide for Care and Use of Laboratory Animal (Pub No. 85-23, revised 1985).

### Acute toxicity and lethality test

The acute toxicity and lethality (LD_50_) of AE in mice (n = 12) was estimated using the method described by Lorke [16]. In stage one of the test, animals received oral administration of one of 10, 100, or 1000 mg/kg (n = 3) of AE and observed for 24 h for number of deaths. Since no death occurred in any of the groups in the first stage of the test, 1600, 2900 and 5000 mg/kg doses of the extract were administered to a fresh batch of animals (n = 1) and no death was recorded within 24 h. A repeat of the test with a fresh batch of animals using the intraperitoneal route also gave the same result. Thus, the oral and ip LD_50 _in mice was found to be greater than 5000 mg/kg.

### Antimicrobial activity test

The inhibitory effect of AE on the growth of strains of *Pseudomonas aeruginosa *and *Staphylococcus aureus *clinically isolated from wounds and identified as earlier described [17] was studied using the cup-plate diffusion method [18]. Briefly, sterile molten agar (20 ml) was seeded with 0.1 ml of standardized broth culture of bacteria (1 × 10^6 ^CFU/ml). When set, four drops (0.02 ml per drop) of each concentration (3.125, 6.2, 12.5, 25, 50, and 100 mg/ml) of the extract were placed in wells (n = 5/plate) bored on the agar by means of a sterile cork borer (6 mm diameter). The plates (n = 5 per test organism) were incubated at 37°C for 24 h after which the inhibition zone diameter (IZD) of each concentration of the extract was measured. Distilled water or gentamicin (0.7 mg/ml) was used as control.

### Topical acute edema of the mouse ear

The effect of AE on acute topical inflammation was evaluated by a modification of the methods of Tubaro *et al *[19] and Atta and Alkofahi [20]. Adult Swiss albino mice (15–25 g) of either sex were divided into three groups of 10 animals. The treatment group received AE (5 mg/ear) applied on the anterior surface of the right ear. Topical inflammation was instantly induced on the posterior surface of the same ear by application of xylene (0.05 ml). Control animals received either distilled water or indomethacin (5 mg/ear). Two hours after induction of inflammation, mice were killed by overdose of ether anesthesia and both ears removed. Circular sections (7 mm diameter) of both the right (treated) and left (untreated) ears were punched out using a cork borer, and weighed. Edema was quantified as the weight difference between the two earplugs. The anti-inflammatory activity was evaluated as percent edema reduction/inhibition in the treated animals relative to control animals [19,21] using the relation;

Edema reduction/inhibition (%) = 100 [(Rt-Lt)/(Rc-Lc)]

Where R_t _= mean weight of right ear plug of treated animals; L_t _= mean weight of left ear plug of treated animals; R_c _= mean weight of right ear plug of control animals; L_c _= mean weight of left earplug of control animals.

### Systemic acute edema of the rat paw

The rat paw edema method [22] was used. Acute inflammation was measured in terms of change in volume of the rat hind paw [23] induced by subplantar injection of agar [24,25]. Animals (n = 5/group) received 200, 400 or 800 mg/kg of AE administered orally. Edema was induced one hour later with agar (0.1 ml) injected into the subplantar region of the right hind paw of the rats. The volume of distilled water displaced by the treated paw was measured before and 1, 2, 3, 4, and 5 h after induction of edema. Control groups received either equivalent volume of the vehicle (distilled water) or indomethacin (100 mg/kg). Inflammation was assessed as the difference between the zero time volume of the treated paw (V_o_) and the volume at the various times (V_t_) after the administration of the phlogistic agent. Percent inhibition of edema [26,27] was calculated using the relation: Inhibition of edema (%) = 100 [1-{(a-x)/(b-y)}]

Where a = mean paw volume of treated rats at various time after egg albumin injection; x = mean paw volume of treated rats before albumin injection; b = mean paw volume of control rats at various time after egg albumin injection; y = mean paw volume of control rats before albumin injection.

### Formaldehyde arthritis test

The effect of the extract on chronic inflammation was assessed using arthritis induced by formaldehyde [28] in rats. On day one, adult Swiss albino rats of either sex received the aqueous extract (200, 400 or 800 mg/kg) administered orally. One hour later, arthritis was induced by subplantar injection of 0.1 ml of 2.5% formaldehyde solution and repeated on day 3. Arthritis was assessed by measuring the volume of distilled water displaced by the paw before induction of arthritis and once every day for ten days, starting from day one, after induction of arthritis. Extract administration was continued once daily for ten days. Control animals received either indomethacin (50 mg/kg) or equivalent volume of vehicle (distilled water). The global edematous response was quantified as the area under the curve (AUC) of the time-course of the arthritic event. The AUC was calculated using the trapezoidal rule. The level of inhibition of arthritis was calculated using the relation: Inhibition of arthritis (%) = [1 - (AUCt/AUCc)] 100

Where AUCc = AUC of the control group; AUCt = AUC of the treated group

### Vascular permeability in mice

The effect of the extract on vascular permeability was assessed in mice using the method of Whittle [29]. Briefly, one hour after oral administration of AE (200 or 400 mg/kg), Evans Blue dye (0.2 ml of a 0.25% solution in Normal saline) was intravenously administered through the tail vein. Control animals received either indomethacin (50 mg/kg) or equivalent volume of vehicle (distilled water). Thirty minutes later, animals received intraperitoneal injection of 1 ml/100 g of acetic acid (0.6% v/v). Treated animals were sacrificed 30 min after acetic acid injection and the peritoneal cavity washed with normal saline (3 ml) into heparinized tubes and centrifuged. The dye content in the supernatant was measured at 610 nm using Spectronic 21D (Milton Roy) spectrophotometer.

### Membrane stabilization activity

#### (i) Preparation of erythrocyte suspension

Fresh whole ox blood (10 ml) was collected, transferred to heparinized centrifuge tubes, centrifuged at 3000 rpm for 5 min, and washed three times with equal volume of normal saline. The volume of the blood was measured and reconstituted as a 40% (v/v) suspension with isotonic buffer solution (10 mM sodium phosphate buffer pH 7.4). The composition of the buffer solution (g/L) was NaH_2_PO_4 _(0.2), Na_2_HPO_4 _(1.15) and NaCl (9.0) [30].

#### (ii) Heat induced haemolysis

The isotonic buffer solution (5 ml) containing 200 and 400 μg/ml of the aqueous extract were put in 4 sets (per concentration) of centrifuge tubes. Control tubes contained 5 ml of the vehicle or 5 ml of 100 ug/ml of hydrocortisone. Erythrocyte suspension (0.05 ml) was added to each tube and gently mixed. A pair of the tubes was incubated at 54°C for 20 min in a regulated water bath. The other pair was maintained at 0–4°C in a freezer for 20 min. At the end of the incubation, the reaction mixture was centrifuged at 1300 g for 3 min and the absorbance (OD) of the supernatant measured at 540 nm using Spectronic 2ID (Milton Roy) spectrophotometer. The level of inhibition of hemolysis was calculated using the relation [30]: Inhibition of hemolysis (%) = 100 [1-{(OD_2_-OD_1_)/(OD_3_-OD_1_)}]

Where OD_1 _= absorbance of test sample unheated; OD_2 _= absorbance of test sample heated; OD_3 _= absorbance of control sample heated

#### (iii) Hypotonicity-induced haemolysis

The hypotonic solution (distilled water) (5 ml) containing 200 or 400 μg/ml of AE was put in 2 pairs (per dose) of centrifuge tubes. Control tubes contained 5 ml of the vehicle (distilled water) or hydrocortisone (0.5 mg/5 ml). Erythrocyte suspension (0.05 ml) was added to each tube and after gentle mixing, the mixtures were incubated for 1 h at room temperature (31°C). After incubation, the reaction mixture was centrifuged for 3 min at 1300 g and the absorbance (OD) of the supernatant measured at 540 nm using Spectronic 21D (Milton Roy) spectrophotometer. The inhibition (%) of haemolysis was calculated using the relation [30]:

Inhibition of haemolysis (%) = 100 [1-{(OD_2_-OD_1_)/(OD_3_-OD_1_)}]

Where OD_1 _= absorbance of test sample in isotonic solution; OD_2 _= absorbance of test sample in hypotonic solution; OD_3 _= absorbance of control sample hypotonic solution

### Leukocyte migration induced by inflammatory stimulus

The effect of AE on leukocyte migration in vivo was studied in rats using the method described by Ribeiro *et al*. [31]. Adult albino rats (150–210 g) of either sex were used. The extract was administered orally at 200, 400, or 800 mg/kg to animals (n = 5/dose). One hour after drug administration, animals received intraperitoneal injection of 1 ml of 2.8% w/v agar. Four hours later, the animals were sacrificed and the peritoneal cavities washed with 5 ml of phosphate buffer saline containing 0.5 ml of 10% EDTA. Total and differential leukocyte counts in the peritoneal wash were taken and the level of inhibition (%) or otherwise of neutrophil and lymphocyte migration was calculated.

### Macrophage phagocytic activity

The effect of the extract on phagocytic activity of mice peritoneal macrophages was studied using the methods described by Rege and Dahanukar [32] and Handel-Fernandez and Lopez [33].

#### (i) Harvesting of mice peritoneal macrophages

Mice selected without sex discrimination were sensitized by intraperitoneal administration of 0.1 ml of 50% peptone. Treated animals received once daily oral administration of AE (200, 400 or 800 mg/kg) for four days. Control animals received distilled water or metronidazole (300 mg/kg). On day four, the animals were sacrificed, and the peritoneal macrophages collected in 10 ml of Hank's solution. The cells were washed by centrifuging at 3000 g for 10 min, and re-suspended in Minimum Enrichment Medium [32].

#### (ii) Preparation of *Candida albicans *suspension

Laboratory strains of *C. albicans *were collected in loops from cultures that were at least 3 days old and had entered the yeast phase. The organisms were washed twice by centrifuging at 2000 g for 10 min in 0.9% saline and a third time in PBS [33].

#### (iii) Macrophage phagocytic activity assay

An assay system containing *C. albicans *suspension (0.1 ml), macrophage suspension (0.3 ml), DMEM (0.3 ml) and Fetal Bovine Serum (0.1 ml) was incubated in a carbon dioxide environment at 37°C for 1.5 h. After incubation, the assay samples were smeared on microscope slides, fixed with ethanol and stained with 10% Giemsa in PBS for 5 min. The slides were washed, air-dried and viewed in a light microscope (×100) under oil of immersion [32]. The number of macrophages with and without ingested *C. albicans *was calculated per high power field. Phagocytic activity was assessed in terms of the proportion of macrophages that ingested *C. albicans *versus free and total macrophage count [34].

### Delayed type hypersensitivity reaction induced by sheep red blood cells in rats

The effect of AE on specific cell-mediated immune responses was evaluated using the delayed hypersensitivity reaction (DTHR) induced by sheep red blood cells (SRBC) in rats.

#### (i) Preparation of sheep erythrocytes

Fresh sheep blood (10 ml) was aseptically withdrawn from the jugular vein of a healthy male sheep and transferred to heparinized tube. The blood samples were centrifuged at 3000 g for 3 min and the pellets washed with equal volume of pyrogen-free normal saline. At the last washing, the red blood cell count (7.3 × 10^6 ^cells/ml) was taken.

#### (ii) Delayed type hypersensitivity reaction (DTHR) test

The method of Doherty [35] was used. Adult Swiss albino rats (110–210 g) of either sex were divided into 5 groups. Each group (n = 4) received 200, 400 or 800 mg/kg of AE administered orally. Control animals received either Levamisole (2.5 mg/kg) [36] or distilled water. On day zero, one hour after AE administration, the rats were sensitized by injecting fresh SRBCs (0.1 ml of 7.3 × 10^6 ^cells/ml) intradermally into the right hind foot paw. Extract administration was continued once daily for 7 days. On day 7, the sensitized animals were challenged by intradermal injection of SRBCs (0.1 ml) into the paw of the left hind foot. The paw size was determined by the volume of distilled water displaced by the paw before and 24 h after challenge. Edema formation was quantified as the difference in the volume of the inflamed paw. The level of inhibition of edema formation was calculated using the relation:

$$\text{Inhibition of edema (\%)}=\text{100}\left[\text{1}-\text{(}\frac{\text{a-x}}{\text{b-y}})\right]$$

Where a = mean paw volume of treated rats after sheep red blood cells challenge; x = mean paw volume of treated rats before sheep red blood cells challenge; b = mean paw volume of control rats after sheep red blood cells challenge; y = mean paw volume of control rats before sheep red blood cells challenge.

### Statistical analysis

Data obtained was analyzed using One Way ANOVA and further subjected to LSD post hoc test. Results are expressed as Mean ± SEM. Difference between Means of treated and control groups was considered significant at *P *< 0.05.

## Results

### Effect of the extract on microbial growth

The extract inhibited the growth of both test organisms only at 50 and 100 mg/ml. The inhibitory effect of the extract was however, lower than that of gentamicin (0.7 mg/ml) (Table 1).

**Table 1:** Antimicrobial activity of the extract

**Extract**	**Concentration (mg/ml)**	**Inhibition Zone Diameter (mm)**	
		
		***Ps. Aeruginosa***	***Staph. aureus***
Control	-	0.0 ± 0.0	0.0 ± 0.0
AE	50	10.0 ± 0.27	10.0 ± 0.32
	100	15.0 ± 0.32	19.3 ± 0.18
Gentamicin	0.7	15.0 ± 0.16	33.0 ± 0.32

AE = Aqueous Extract; n = 5; Diameter of cork borer = 6 mm; Control = DMSO (Dimethylsulphoxide)

### Effect of the extract on topical acute edema

Topical application of the extract significantly (*P *< 0.05) inhibited edema induced by xylene in the mouse ear provoking an inhibitory effect as high as 57%. The same dose of indomethacin used as the reference drug caused 66.7% inhibition (Table 2).

**Table 2:** Effect of extract on topical acute edema of the mouse ear

**Extract**	**Dose (mg/ear)**	**Edema (mg)**	**Inhibition (%)**
AE	5.0	1.29 ± 0.52^a^	57.0
Indomethacin	5.0	1.00 ± 0.53^a^	66.7
Control	-	3.00 ± 0.23	-

^a^*P *< 0.05 compared to control (ANOVA; LSD post hoc); AE = Aqueous extract;

Values of ear edema shown are Mean ± SEM (n = 10).

### Effect of the extract on systemic acute edema

Oral administration of the extract significantly (*P *< 0.05) suppressed the development of acute edema of the rat paw induced by agar at the 3 doses tested. The extract evoked a non-dose-related effect with the lowest dose (200 mg/kg) exhibiting the highest inhibition which was also better than that of indomethacin (100 mg/kg) (Table 3).

**Table 3:** Effect of extract on agar-induced acute edema of the rat paw

**Extract**	**Dose (mg/kg)**	**Edema (ml)**					
		
		**0.5 h**	**1 h**	**2 h**	**3 h**	**4 h**	**5 h**
AE	200	0.14 ± 0.02 (33.33)	0.14 ± 0.02* (44.00)	0.15 ± 0.02* (51.61)	0.14 ± 0.02* (58.82)	0.19 ± 0.07* (57.78)	0.20 ± 0.07* (58.33)
	400	0.16 ± 0.02 (28.81)	0.16 ± 0.02* (36.0)	0.18 ± 0.03* (41.94)	0.18 ± 0.04* (47.06)	0.26 ± 0.08* (42.22)	0.35 ± 0.05 (27.08)
	800	0.16 ± 0.02 (28.81)	0.18 ± 0.03* (28.00)	0.23 ± 0.06 (25.81)	0.26 ± 0.07 (23.53)	0.31 ± 0.06 (31.11)	0.36 ± 0.06 (25.0)
Indomethacin	100	0.16 ± 0.01 (28.81)	0.18 ± 0.03* (28.00)	0.18 ± 0.03* (41.94)	0.21 ± 0.03* (38.24)	0.29 ± 0.04 (35.56)	0.30 ± 0.04* (37.50)
Control	-	0.21 ± 0.01	0.25 ± 0.00	0.31 ± 0.01	0.34 ± 0.01	0.45 ± 0.03	0.48 ± 0.01

*Reduction in edema significant at *P *< 0.05 compared to control (ANOVA; LSD post hoc). Values of edema shown are Mean ± SEM (n = 5); AE = Aqueous extract; Values in parenthesis represent percent inhibition of edema calculated relative to the Control.

### Effect of the extract on chronic inflammation of the formaldehyde arthritis

Daily oral administration of the extract effectively suppressed the early stage of development of formaldehyde arthritis (data not shown). However, the extract was not effective in inhibiting the later stages of the arthritis event and hence did not reduce the global edematous response due to arthritis (Table 4).

**Table 4:** Effect of extract on global edematous response to arthritis induced by formaldehyde

**Extract**	**Dose (mg/kg)**	**AUC (ml/day)**	**Inhibition (%)**
AE	200	2.93 ± 0.41	NI
	400	2.66 ± 0.09	NI
	800	2.69 ± 0.52	NI
Indomethacin	100	1.85 ± 0.23	28.84
Control	-	2.60 ± 0.06	-

Values of AUC shown are Mean ± SEM (n = 5); AE = Aqueous extract;

NI = No inhibition

### Effect of the extract on vascular permeability

Oral administration of the extract evoked a mild dose-related inhibition of vascular permeability experimentally induced by acetic acid in mice. Indomethacin exhibited a greater inhibition than 400 mg/kg dose of the extract (Table 5).

**Table 5:** Effect of extract on acetic acid-induced vascular permeability

**Extract**	**Dose (mg/kg)**	**Absorbance (610 nm)**	**Inhibition (%)**
AE	200	0.38 ± 0.04	5
	400	0.36 ± 0.02	10
Indomethacin	50	0.20 ± 0.01	50
Control	-	0.40 ± 0.02	-

AE = Aqueous extract; Values of absorbance shown are Mean ± SEM (n = 5)

### Effect of the extract on membrane stabilization

The extract protected the erythrocytes against heat- and hypotonicity-induced haemolysis in a concentration-related manner. The protective effect was greater on haemolysis induced by heat than that caused by hypotonicity (Table 6).

**Table 6:** Effect of extract on heat- and hypotonicity-induced haemolysis of red blood cells

**Extract**	**Concentration (μg/ml)**	**Heat-induced haemolysis (Inhibition %)**	**Hypotonicity-induced haemolysis (Inhibition %)**
AE	50	14	12
	100	67	16
	200	120	23
	400	400	45
Hydrocortisone	50	50	97.5

n = 5; AE = Aqueous extract; Inhibition (%) of haemolysis shown was derived from absorbance values

### Effect of the extract on cell migration

Oral administration of the extract increased total leukocyte and neutrophil counts at both dose levels. However, the cell counts decreased in a dose-related manner (Table 1). The extract also caused a dose-related increase in the macrophage count that was significant (*P *< 0.05) at the 800 mg/kg dose (Table 7).

**Table 7:** Effect of extract on leukocyte and macrophage migration *in vivo*

**Extract**	**Dose (mg/kg)**	**Leukocyte Count**		**Macrophage Count (Cells/High Power Field)**
			
		**TLC × 10^3 ^(Cells/ml)**	**Neutrophil Count (%)**	
Control	**-**	3.30 ± 1.6	55.0 ± 5.5	6.33 ± 0.88
AE	200	10.9 ± 3.2 (230.30)	73.0 ± 9.6 (32.73)	7.97 ± 0.38 (26.5)
	400	10.0 ± 3.2 (203.03)	69.7 ± 12.0 (26.73)	9.53 ± 1.92 (50.8)
	800	3.97 ± 2.4 (20.30)	52.7 ± 99.0 (-4.18)	14.20 ± 2.62* (125.4)
Levamisole	2.5	1.77 ± 0.3 (-46.36)	40.0 ± 6.2 (-27.27)	NT
Metronidazole	300	NT	NT	9.75 ± 0.9 (54.03)

TLC = Total Leukocyte Count; Macrophage Count = Total number of cells per high power field; Values in parenthesis represent the percent migration of cells; NT = Not tested; AE = Aqueous extract; *P < 0.05 compared to Control (*ANOVA; LSD post hoc*)

### Effect of the extract on phagocytosis

The extract caused a dose-related increase in the number (and proportion) of macrophages with ingested *C. albicans*. The increase was significant (*P *< 0.05) at the 800 mg/kg dose. It also reduced the proportion of free cells in a dose-related manner (Table 8).

**Table 8:** Effect of extract on phagocytic activity of peritoneal macrophages

**Extract**	**Dose (mg/kg)**	**Macrophage Count**	**Macrophage phagocytic activity**			
			
			**Free Cells**	**Proportion of free cells (%)**	**Cells with ingested *C. albicans***	**Proportion of cells with ingested *C. albicans*(%)**
Control	-	6.33 ± 0.88	5.3 ± 0.88	84.13	1.00 ± 0.0	15.87
AE	200	7.97 ± 0.38	6.1 ± 0.29	76.53	1.87 ± 0.13	23.46
	400	9.53 ± 1.92	7.3 ± 1.57	76.84	2.20 ± 0.42	23.16
	800	14.20 ± 2.62*	9.3 ± 0.75*	65.49	6.10 ± 3.02*	42.96
Metronidazole	300	9.75 ± 0.9	4.25 ± 0.14	43.59	5.50 ± 0.43*	56.41

AE = Aqueous Extract; Values of macrophage count shown are Mean ± SEM calculated per high power field (n = 3 determinations); Values in parenthesis represent percentage increase in number of macrophages calculated relative to control; Proportion (%) of free cells (cells without *C. albicans*) and cells with ingested *C. albicans *were calculated relative to the total number of macrophages; *P < 0.05 compared to control (*ANOVA; LSD post hoc*).

### Effect of the extract on SRBC-induced DTHR

The extract significantly (*P *< 0.05) inhibited DTHR induced by SRBCs in rats in a dose-related manner. The magnitude of inhibition evoked by the extract was higher than that exhibited by levamisole (Table 9).

**Table 9:** Effect of extract on SRBC-induced DTHR

**Extract**	**Dose (mg/kg)**	**Paw size (ml)**	**Inhibition (%)**
Control	-	0.40 ± 0.05	-
AE	200	0.25 ± 0.03	37.5
	400	0.17 ± 0.04*	57.5
	800	0.18 ± 0.04*	55.0
Levamisole	2.5	0.28 ± 0.06	30.0

n = 4; AE = Aqueous extract; *P < 0.05 (*ANOVA; LSD post hoc*); SRBCs = Sheep red blood cells; DTHR = Delayed type hypersensitivity reactions

## Discussion

Furuncles or boils develop as a consequence of bacterial infection and treatment with antimicrobial agents eases the discomfort by eradicating the causal organism. Assessment of the antimicrobial effect of *A. montanus *showed that the aqueous root extract demonstrated moderate antimicrobial activity against clinically isolated strains of *P. aeruginosa *and *S. aureus*, the causal organisms mostly implicated in boils [11]. The effect of the extract was higher against *S. aureus *than *P. aeruginosa *which is an indication of greater susceptibility of the former implicated as the major causal organism of boils [11]. However, the magnitude of the antimicrobial activity seems not to suggest that the roots derive their acclaimed potency in treatment of furuncles from antimicrobial effect. Although treatment of boils with antimicrobial agents may be curative in this instance (depending on the size of the boil), anti-inflammatory agents provide instant relief from the pain and other discomfort caused by the inflammatory reactions invoked by the causal organisms. Our study showed that topical and systemic administration of the extract resulted to inhibition of the development of acute edema. Topical application of the extract is consistent with traditional use and the relief obtained in the treatment of boils may also derive from suppression of acute inflammatory reactions. Thus, phytochemical constituents of the roots are likely capable of permeating inflamed skin to exert antimicrobial and anti-inflammatory effects.

The acute inflammatory reaction is a physiological characteristic of vascularized tissues [37] and increased vascular permeability associated with it is known to cause exudation of fluid rich in plasma proteins including immunoglobulins, coagulation factors and cells into the injured tissues with subsequent edema at the site [38,39]. Increased vascular permeability is a major feature of acute inflammation [40] and results from contraction and separation of endothelial cells at their boundaries to expose the basement membrane which is freely permeable to plasma proteins and fluid [41]. In this study, the extract moderately inhibited increased vascular permeability induced by acetic acid in the mouse peritoneum. Acetic acid causes an immediate sustained reaction that is prolonged over 24 h [12,40] and the inhibition caused by the extract suggests it may suppress exudation and its consequences and thus modulate the magnitude of the inflammatory response associated with boils. On the proliferative phase of inflammation, the extract was not effective in ameliorating the overall outcome of formaldehyde arthritis event notwithstanding the fact that it inhibited the early stages (which is likely an extension of its effect on acute inflammation). The usefulness of the extract may be limited to acute inflammatory conditions such as obtained in boils and which is consistent with the ethnomedicinal use of the plant roots. As such, the extract may not offer any relief when used in disorders of chronic inflammation such as rheumatoid arthritis.

Another key aspect of the inflammatory response is cellular infiltration due to the pivotal role played by leukocytes in inflammation. These cells are found in boil cavities where the dead cells and necrotic tissues form components of pus [13]. As part of their defensive roles during inflammation, these cells release their lysosomal contents such as bactericidal enzymes and proteases, at sites of inflammation which cause further tissue damage and inflammation [42]. Stabilization of the membranes of these cells inhibits lysis and subsequent release of the cytoplasmic contents which in turn limits the tissue damage and exacerbation of the inflammatory response. *In vitro *assessment of the effect of the extract on membrane stabilization showed that it inhibited heat- and hypotonicity-induced lysis of ox red blood cells. Although we do not know the precise mechanism of membrane stabilization in this case, direct interaction of constituents of the extract with membrane components such as proteins seems most probable. Membrane proteins are largely responsible for the physical properties of the cell membrane and may contribute to the regulation of the volume and water content of cells by controlling the movement of sodium and potassium ions [43] through the protein channels which make up ion channels in the cell membrane [13]. Since pathological conditions can alter surface-volume ratio of the cell through loss of membrane surface or gain in volume [43], the physical integrity of the treated cell membranes may have been enhanced by the extract (through a direct protective interaction with the membrane proteins) to hinder cell lysis including that caused by products such as those of the complement system involved in the inflammatory response cascade and hypotonic solutions that cause the cell to swell and rupture [43]. The stability of biological membranes is also affected by reactive oxygen species and we have in an earlier study (data not shown) shown that the extract exhibited antioxidant effect by inhibiting lipid peroxidation induced by CCl_4 _and ferrous sulphate in the rat kidney and liver [44]. This action suggests that the extract may enhance membrane stability through antioxidant effect and protect the tissues from damage due to injurious products of oxidative stress implicated in the pathogenesis of inflammatory disorders [45]. Furthermore, inhibition of cell migration or infiltration is associated with anti-inflammatory effect. Cellular migration involves a sequence of well-documented events [46-49]. Expression of chemoattractant factors and response of these cells to chemotactic signals are central to their migration. In addition to enhancing neutrophil migration, chronic administration of the extract to mice increased the population of macrophages which migrated to the peritoneum in response to stimulation. These independent but related tests confirm that the root extract may stimulate or enhance cellular migration. However, the extent of interaction of the extract with the processes associated with cell recruitment is not known to us. Like neutrophils, macrophages also migrate in response to chemotactic factors and the enhancement of their migration by the extract may involve similar mechanisms. Since inhibition of cell migration is known to contribute to anti-inflammatory activity, enhancement or stimulation of migration by the extract runs counter to the anti-inflammatory activity. Our result also showed that increasing the dose of the extract reduced the extent or magnitude of cell migration which suggests that the extract may as well inhibit cell migration under acute inflammatory response. However, the occurrence of this effect only at high doses not likely employed in traditional use of the roots in treatment of boils indicates that inhibition of cell migration may not contribute to the anti-inflammatory effect and most unlikely to play any role in the relief obtained. Thus, although anti-inflammatory effect may relieve the discomfort caused by boils, constituents of roots of this plant may owe part of their action in furuncles to a more fundamental mechanism the magnitude of which may be dependent on the extent of cell migration which they appear to enhance.

In furtherance of their defensive roles at sites of infection, leukocytes engage in phagocytosis to contain the microbial invasion. Evaluation of the effect of the extract on phagocytosis showed that it enhanced the phagocytic activity of mouse peritoneal macrophages quantified as increase in the number of macrophages that ingested *C. albicans*. The phagocytic activity of these cells also results to the generation of the necrotic tissue debris and dead cells known to be components of pus [13] and may be the major mechanism responsible for the acceleration of pus formation seen when the root is used in the treatment of boils. In addition to phagocytic activity, we also studied the effect of the extract on specific immune responses mediated by the T lymphocytes which is important for host defense against intracellular parasites [50]. Our results showed that the extract inhibited the development of DTHR induced by SRBCs in rats. The DTHR is characterized by a heavy infiltration of T cells and macrophages [14] in response to antigenic stimulation which in this case was the SRBCs. Inhibition of T lymphocyte infiltration seems to contradict the enhancement of leukocyte migration earlier noted. The disparity in the effect of the extract on cell types may lie in the mechanisms involved in the migration of these different species of leukocytes. Neutrophils migrate in response to chemoattractant gradient and chemotactic factors while the migration of lymphocytes is stimulated by specific antigens, mitogens and other non-specific factors produced by lymphocytes [50]. Lymphocytes though motile cells, do not respond to most of the chemotactic factors that act on polymorphonuclear leukocytes and macrophages [50]. Consequently, we may safely assume that the extract promotes the expression of chemotactic/chemoattractant factors although we do not know its extent of interaction with processes involved in antigen recognition which is the initial stimulus for T cell activation [14]. This makes it also most likely that phytochemical constituents of the root responsible for its effectiveness in furuncle treatment may largely interfere with or suppress immune processes with other mechanisms such as antimicrobial and anti-inflammatory activities playing supportive roles.

The phytochemical constituents responsible for these activities are yet to be identified. The pharmacological actions of the saponins [8] and the gammaceranes- acanthusol and its 3-O-β-D-glucopyranoside [9] isolated form this plant are not known as well as their roles in the activities studied. Phytochemical analysis of the root extract revealed an abundant presence of alkaloids and carbohydrates while saponins, glycosides, and terpenoids occurred moderately. The relatively high presence of alkaloids and carbohydrates suggests that these constituents may largely account for the pharmacological activities of the plant roots.

Oral and intraperitoneal administration of the extract to mice caused no deaths at doses ranging from 10–5000 mg/kg suggesting an oral and i.p LD_50 _greater than 5000 mg/kg. The high LD_50 _value implies a remote risk of acute intoxication and a high degree of relative safety independent of route of administration. Although the roots are used externally, the relative safety is obviously advantageous since systemic administration and or systemic absorption of the root extract from site of topical application (as employed in ethnomedicine) may pose no risk of acute toxicity.

## Conclusion

The results of this study showed that the effectiveness of roots of *A. montanus *in the treatment of boils may derive largely from enhancement of cellular migration as well as phagocytic activity which are involved in the non-specific defense of the body from infection while antimicrobial and anti-inflammatory (mediated through inhibition of increase in vascular permeability and membrane stabilization) activities may play contributory roles. The results further provide a rationale for the use of the roots in treatment of boils. Phytochemical constituents such as alkaloids and carbohydrates may be responsible for these activities. Biological activity-guided fractionation of the aqueous extract is ongoing for isolation of the anti-inflammatory principle.

## Competing interests

The authors declare that they have no competing interests.

## Authors' contributions

COO designed the study, performed some of the pharmacology experiments, analysed the data and drafted the manuscript; PAA participated in the experimental design, coordinated the experimental work and preparation of the manuscript; NJO carried out studies on the phagocytic activity; TCO participated in some of the pharmacology experiments and writing of the manuscript while ACN carried out some aspects of the anti-inflammatory activity experiments. CSN participated in some of the pharmacology experiments and writing of the manuscript. All authors read and approved the final manuscript.

## Pre-publication history

The pre-publication history for this paper can be accessed here:



## References

[B1] Huxley AJ (1992). *Acanthus montanus *plant description and geographical distribution. The New Royal Horticultural Society dictionary of gardening.

[B2] Igoli JO, Tor-Anyiin TA, Usman SS, Oluma HOA, Igoli PN, Singh VK, Govil SH, Singh S (2004). Folk medicines of the lower Benue valley in Nigeria. Recent Progress in Medicinal Plants Ethnomedicine and Pharmacognosy II.

[B3] Didie VS (2005). Plant remedies from the Democratic Republic of Congo. Medicinal Plants Database, African Research Museum.

[B4] Ibe AE, Nwufo MI (2005). Identification, collection and domestication of medicinal plants in southern Nigeria. African Dev.

[B5] Adeyemi OO, Okpo SO, Young-Nwafor CC (1999). The relaxant activity of the methanolic extract of *Acanthus montanus *on intestinal smooth muscles. J Ethnopharmacol.

[B6] Adeyemi OO, Okpo SO, Okpaka O (2004). The analgesic effect of the methanolic extract of *Acanthus montanus*. J Ethnopharmacol.

[B7] Asongalem EA, Foyet HS, Ekobo S, Dimo T, Kamtchouing P (2004). Anti-inflammatory, lack of central analgesia and antipyretic properties of *Acanthus montanus *(Ness) T. Anderson. J Ethnopharmacol.

[B8] Anam EM (1997). Novel triterpenoid glycoside and triterpenoid acid from the root extract of *Acanthus montanus *(Acanthaceae). Ind J Chem Sect B.

[B9] Anam EM (1997). Pentacyclic triterpenoids from *Acanthus montanus *(Acanthaceae). Ind J Chem Sect B.

[B10] Igoli JO, Ogaji OG, Tor-Anyiin TA, Igoli PN (2005). Traditional medicine practice amongst the Igede people of Nigeria. Part II. African J Trad Compl Altern Med.

[B11] Sleigh JD, Pennington TH, Lucas SB, MacSween RMN, Whaley K (2001). Microbial infections. Muir's Textbook of Pathology.

[B12] Whaley K, Burt AD, MacSween RMN, Whaley K (1996). Inflammation, healing and repair. Muir's Textbook of Pathology.

[B13] Guyton CA, Hall JE (2000). Textbook of Medical Physiology.

[B14] Mowat AM, Whaley K, MacSween RMN, Whaley K (1996). The structure and function of the immune system. Muir's Textbook of Pathology.

[B15] Harbone JB (1984). Phytochemical Methods: a guide to modern techniques of plant analysis.

[B16] Lorke D (1983). A new approach to practical acute toxicity testing. Arch Toxicol.

[B17] Okoli CO, Akah PA, Okoli AS (2007). Potentials of leaves of *Aspilia africana *(Compositae) in wound care: an experimental evaluation. BMC Compl Altern Med.

[B18] Lovian V (1980). Antibiotics in Laboratory Medicine.

[B19] Tubaro A, Dri P, Delbello G, Zilli C, Della LR (1985). The croton oil ear test revisited. Agents Actions.

[B20] Atta AH, Alkohafi A (1998). Antinociceptive and anti-inflammatory effects of some Jondanian medicinal plants extracts. J Ethnopharmacol.

[B21] Asuzu IU, Sosa S, Della LR (1999). The anti-inflammatory activity of *Icacina trichantha *tuber. Phytomed.

[B22] Winter CA, Risley EA, Nuss GW (1962). Carrageenin-induced edema in the hind paw of the rat as an assay for anti-inflammatory drugs. Proc Soc Exp Biol Med.

[B23] Backhouse N, Delporte C, Negrete R, Salinus P, Pinto A, Aravena S, Cassels BK (1996). Anti-inflammatory and antipyretic activities of *Cuscuta chilensis, Cestrum parqui*, and *Psolarea glandulosa*. Int J Pharmacog.

[B24] Okoli CO, Akah PA, Nwafor SV, Anisiobi AI, Ibegbunam IN, Erojikwe O (2007). Anti-inflammatory activity of hexane leaf extract of *Aspilia africana *C.D. Adams. J Ethnopharmacol.

[B25] Iwueke AV, Nwodo OFC, Okoli CO (2006). Evaluation of the anti-inflammatory and analgesic activities of *Vitex doniana *leaves. Afr J Biotech.

[B26] Ahmed MM, Qureshi S, Al-bekairi AM, Shah AH, Rao RM, Qazi NS (1993). Anti-inflammatory Activity of *Caralluma tuberculata *alcoholic extract. Fitoterapia.

[B27] Perez GRM (1996). Anti-inflammatory activity of *Ambrosia artemisiaefolia *and *Rheo spathaceae*. Phytomed.

[B28] Seyle H (1949). Further studies concerning the participation of adrenal cortex in the pathogenesis of arthritis. Brit Med J.

[B29] Whittle BA (1964). The use of changes in capillary permeability in mice to distinguish between narcotic and non-narcotic analgesics. Brit J Pharmacol.

[B30] Shinde UA, Phadke AS, Nari AM, Mungantiwar AA, Dikshit VJ, Saraf MN (1999). Membrane stabilization activity – a possible mechanism of action for the anti-inflammatory activity of *Cedrus deodora *wood oil. Fitoterapia.

[B31] Ribeiro RA, Flores CA, Cunha FQ, Ferreira SH (1991). IL-8 causes *in vivo *neutrophil migration by a cell dependent mechanism. Immunol.

[B32] Rege NN, Dahanukar SA (1993). Quantitation of microbicidal activity of mononuclear phagocytes: an in vitro technique. J Postgrad Med.

[B33] Handel-Fernandez ME, Lopez DM, Paulnoch D (2004). Isolation of macrophages from tissues, fluids and immune response sites. Macrophages.

[B34] Stites DP, Stites DP, Stob JO, Wells JV (1987). Clinical lab methods for detection of cellular immune function. Basic and Clinical Immunology.

[B35] Doherty NS (1981). Selective effect of immunosuppressive agents against the delayed type hypersensitivity response and humoral response to sheep red blood cells in mice. Agents Actions.

[B36] Sharma ML, Singh B, Chandan BK, Khajuria A, Kaul A, Bani S, Banerjee SK, Gambhir SS (1996). Action of some flavonoids on specific and non-specific immune mechanism. Phytomed.

[B37] Rang HP, Dale MM, Ritter JM (1999). Textbook of Pharmacology.

[B38] Burt AD, Smith TW, MacSween RMN, Whaley K (1996). Healing and repair. Muir's Textbook of Pathology.

[B39] Cotran RS, Kumar V, Collins T (1999). Robbin's Pathological Basis of Disease.

[B40] Hiley P, Barber PC (2000). Acute inflammation. Home page of the Pathology Dept Medical School, University of Birmingham, UK.

[B41] Brown JN, Roberts J, Gilman AG, Hardman JG, Limbird LE (2001). Histamine, bradykinin, and their antagonists. Goodman and Gilman's The Pharmacological Basis of Therapeutics.

[B42] Chou CT (1997). The anti-inflammatory effect of *Tripterygium wilfordii *Hook F on adjuvant-induced paw edema in rats and inflammatory mediators release. Phytother Res.

[B43] Rowman RM, MacSween RMN, Whaley K (1996). The blood and bone marrow. Muir's Textbook of Pathology.

[B44] Okoli CO, Njoku OU, Ononogbu IC, Ezike AC, Akah PA (2007). Medicinal plants and antioxidant activity. Principles and Practice of Ethnomedicine (African Approach).

[B45] Das BS, Patnalk JK, Mohanty S, Salpathy SK, Mishra SK, Mohanty D, Bose TK (1988). Enhanced plasma lipid peroxidation in falciparum malaria. Ind J Clin Biochem.

[B46] Lawrence MB, Springer TA (1991). Leukocytes roll on a selectin at physiologic flow rates: distinction and prerequisite for adhesion through integrins. Cell.

[B47] Von Andrian UH, Chambers JD, McEvoy LM, Bargatze RF, Drfors KE, Butcher EC (1991). Two-step model of leukocyte-endothelial cell interaction inflammation: distinct roles for LECAM-1 and the leukocyte 2-integrins *in vivo*. Proc Natl Acad Sci USA.

[B48] Konstantopoulos K, Mclintire LV (1996). Effects of fluid dynamics on vascular cell adhesion. J Clin Invest.

[B49] Wagner JG, Roth AR (2000). Neutrophil migration mechanisms, with an emphasis on the pulmonary vasculature 1. Pharmacol Rev Online.

[B50] Gallin JI, Paul WE (1993). Inflammation. Fundamental Immunology.

